# Experimental Investigation to Study the Feasibility of Fabricating Ultra-Conductive Copper Using a Hybrid Method

**DOI:** 10.3390/ma14195560

**Published:** 2021-09-25

**Authors:** Mahesh K. Pallikonda, Taysir H. Nayfeh

**Affiliations:** Washkewicz College of Engineering, Cleveland State University, Cleveland, OH 44115, USA; t.nayfeh@csuohio.edu

**Keywords:** CNTs, electrical conductivity, nanocomposites, electrical conductors, ballistic conductors, Cu/CNT composites

## Abstract

Ultra-conductive copper (UCC) has an enormous potential to disrupt the existing electrical and electronic systems. Recent studies on carbon nanotubes (CNTs), a new class of materials, showed the ballistic conductance of electricity. Researchers around the world are able to demonstrate ultra-conductivity in micro- and millimeter-length sections using various processing techniques by embedding CNTs in the copper matrix. Although multiple methods promise the possibility of producing copper-based nanocomposites with gains in electrical conductivity, thus far, scaling up these results has been quite a challenge. We investigated a hybrid method of both hot-pressing followed by rolling in order to produce UCC wire. Cu/CNT billets of 1/10%, 1/15%, and 1/20% were hot-pressed and the conductivity results were compared to a hot-pressed pure copper billet. Our results indicated that this method is not a viable approach, as the gains in electrical conductivity are neutralized, followed by attenuation of the wire cross-section.

## 1. Introduction

Today, the world is heavily dependent on electricity in all aspects of life, from lighting a house to lighting the International Space Station and running a car on Earth to running a rover on Mars. Electricity and electronics are so integrated in our everyday lives that it is difficult to imagine the world without them. Not limited to current applications, more and more applications are coming forward every day, owing to the growing global electrification and advances in electrical and electronic technologies. Although the demand for electricity exceeds the supply [1], newer methods of harvesting electrical power at large are disrupting the world every day [2]. Apart from the generation of electricity, electrification also involves the transmission and distribution of electricity, which demands an efficient conductor. 

Traditionally, copper and aluminum are the most widely used electrical conductors. Aluminum is used for the transmission of electricity from power grids to substations and transformers. Copper, on the other hand, is used in appliances, house/industrial wiring, and other electrical connectors. Since the invention of voltaic cells, copper has played a pivotal role in electrical conduction. Copper, being a non-precious metal and having a wide range of properties, is the best material for electrical conduction. Copper is by far considered the most commonly used metal for electrical applications. According to the 2021 U.S. Geological Survey Mineral Commodities, 21% of mined copper is used for electrical or electronic products directly [3]. Meanwhile, according to the Copper Development Association, more than half of the copper produced is used for electrical or electronic applications [4]. In 1914, the United States Circular of the Bureau of Standards determined the international annealed copper standard (IACS) as 1.7241 μΩ·cm at 20 °C [5]. This is the value of the electrical resistivity of annealed copper. This value is still in effect, and IACS electrical conductivity is 58 × 10^6^ S/m. Owing to the technological advancements in purifying and processing copper, oxygen-free high-purity copper guarantees 102% IACS. On the other hand, the electrical conductivity of fully cold worked copper is only 5.63 × 107 S/m, which corresponds to 97% IACS. We used IACS representation in this report to avoid misinterpretations with standard annealed copper, oxygen-free copper, and/or other forms of copper metal. Not much development has been made in this field since the earliest conductors (copper). As we have entered into new age of technology, where electricity and electronics are used almost in all aspects, better conductors that are capable of carrying higher currents and low resistance are in demand.

Collins et al. [6] observed that in multi-walled carbon nanotubes (MWCNTs), all shells contribute to electrical conduction. This breakthrough discovery opened the channels for developing ultra-conductive materials by using MWCNTs as a highly conductive filler material. Frank et al. [7] reported the ability to quantize the conductance of MWCNTs and observed a high stable current density of >10^7^. They reported that nanotubes conduct electricity ballistically with no heat loss. Wei et al. [8] also recorded the ability of MWCNTs to carry electrical current of a magnitude higher than 10^9^ A/cm^2^ at elevated temperatures of 250 °C. They also reported that nanotubes show no observable failure and no measurable change in the resistance detected. Li et al. [9] reported, in their experimental observations, that MWCNTs have the capability to carry higher currents at a low bias voltage with perfect ohmic contacts. They reported that the behavior of MWCNTs is due to quasi-ballistic conductance of the inner walls of the CNT. Their experimental results showed higher conductance of MWCNT compared to the theoretical values of single-walled carbon nanotubes (SWCNTs). Hjortstam et al. [10], in their famous article “Can we achieve ultra-low resistivity in carbon nanotube-based metal composites?”, explained the possible challenges in the paths of developing ultra-conductive materials. This article also guides researchers in the possible ways to work toward achieving ultra-conductive conductors.

Nayfeh et al. [11] reported electrical conductivity of 113% IACS by copper nanocomposites produced by using the die-casting method. These results encourage researchers in pursuit of developing nanocomposites that exhibit higher electrical conductivity. Furthermore, other methods for fabricating ultra-conductive conductors have also been investigated [12]. Chen et al. [13], investigating a specific electrolytic co-deposition process, showed an increase in the electrical conductivity of copper/CNT composites by 200% of copper. Many others follow this route for fabricating copper/CNT composites for developing ultra-conductive materials. Cambridge University researchers are developing methods to produce copper/CNT composites by developing CNT–fiber bundles and infiltrating them into copper by using vapor deposition or electrodeposition. Recently, researchers from Shanghai Jiao Tong University [14] demonstrated the ability to produce ultra-conductive copper by embedding graphene into the copper. Graphene is applied on both sides of copper, and thus copper is sandwiched in between graphene, and a stack of such layers is pressed by creating an electron path channel. They showed that the material is able to measure 116% IACS. 

Researchers have been able to demonstrate ultra-conductivity, but the results are not consistent, and the length of the ultra-conductive zones are limited to the ranges of millimeter-long sections [11,12,13,14]. Although multiple methods are being investigated, thus far, scaling up these results has been quite a challenge [12,15]. In this paper, we report the experimental findings of using a hybrid method developed in order to synthesize copper/CNT nanocomposites with enhanced electrical conductivity. 

## 2. Materials and Methods

The CNTs used in this study were MWCNTs, obtained from Applied Sciences Inc., Cedarville, Ohio. The CNTs were functionalized with magnesium, as described in U.S. Patent # 8,347,944. Copper powder of 99.5% purity was supplied by Alfa Aesar Inc., USA (lot no.: H05U037). 

The magnesium functionalization process involved the mixing of anhydrous MgCl_2_ with deionized water in a ratio of 1:4 by weight to form MgCl_2_(H_2_O)_x_. Graphitized MWCNTs (25% (*w*/*w*)) were added to the MgCl_2_(H_2_O)_x_ solution. This suspension was rigorously agitated using a mechanical agitator (Cole-Parmer, Vernon Hills, IL, USA) at 150 rpm for 6 h. Following mechanical stirring, the MgCl_2_(H_2_O)_x_/CNT suspension was agitated by the 20 kHz ultrasonication technique. The 20 kHz ultrasonication was performed with a solid probe (25 mm diameter, titanium alloy) (Sonics & Materials Inc., Newtown, CT, USA) connected to a 20 kHz oscillator (750 watt; Vibra-cell VCX 750, Sonics & Materials Inc., Newtown, CT, USA) for 6 h. The sonicator was operated in a pulsed mode, 10 s on and 20 s off.

Following ultrasonication, the slurry of CNTs and MgCl_2_ was heat-treated in two phases. During the Phase 1 heat treatment, the slurry was held at an elevated temperature of 200 °C for 4 h in a Isotemp Vacuum Oven 282A. The resultant product after the first stage of heating was a dense material, which was broken down into smaller fragments and prepared for the Phase 2 heat treatment. Phase 2 heating was conducted in a high-vacuum chamber. A two-step heat treatment method was programmed for this operation. In step one, the temperature of the furnace was increased at a rate of 5 °C per minute until it reached 300 °C and was held for 1 h; this was necessary for the MgCl_2_ to decompose into magnesium and chlorine. After holding for an hour, step two heating took place, where the temperature of the furnace was programmed to increase until it reached 900 °C at 20 °C per minute. The decomposed chlorine evaporated during this phase. 

The material obtained after the second stage of heating was a softer material and easy to sift. The final powder-like product after sifting was the CNT precursor material, which was used in the hot-pressing operation.

The CNT precursor material obtained after magnesium functionalization was mixed with the copper powder at different ratios. The CNT precursor material was mixed with copper powder in the ratios of 0%, 1/10% (*w*/*w*), 1/15% (*w*/*w*), and 1/20% (*w*/*w*). The mixtures of different concentrations were hot-pressed at 750 °C with a pressing pressure of 2000 psi. The billet obtained after hot-pressing had a diameter of φ15 mm and a height of 10 mm. The billets were later subjected to the rolling operation. The φ15 mm billet was rolled down to φ2 mm and φ1 mm wires. Furthermore, the φ1 mm wire was rolled down to a 0.1 mm thickness and a 2.74 mm-wide ribbon. A commercially available oxygen-free billet of φ15 mm billet was also rolled for calibrating our test equipment. This was essential to eliminate the errors in measurements. Table 1 describes the list of billets and the concentration of CNTs.

Furthermore, in this current study, the electrical conductivity of copper was measured using the four-point resistivity measurement technique. The four-point resistivity measurement technique involves flowing a fixed amount of current between the outer two probes/pins and measuring voltage is measured between the two inner probes/pins. The resultant electrical resistivity is calculated using the measured voltage and the current. The Keithley data acquisition system (Tektronix Inc., Beaverton, OR, USA), consisting of a Keithley ultra-sensitive current source series 6200 model 6221 and a Keithley Nanovoltmeter model 2182A, was used for this purpose. A 100 mA current was applied to the wire and the voltage measurements were recorded. The voltage measurements were recorded for each 5 cm length of wire. Equations (1)–(5) were used in computing the mass conductivity of the wire using the voltage measurements. The mass conductivity provided the true electrical conductivity of the sample by eliminating the density factor. From our density analysis, we determined that the density of the hot-pressed billets was lower than the commercially available copper rods/wire. Table 2 shows the theoretical and measured densities of the billets. Furthermore, Equation (4) mitigates the effect of temperature on the measured resistance by normalizing the readings to 20 °C. The resistance obtained from Equation (4) is the effective resistance of the wire at 20 °C.

(1) $$R=\frac{V}{I}$$

(2) $$R=\frac{R_{T}}{1+\alpha \left(T-20\right)}$$

(3) $$\rho_{v}=\frac{RA}{L}$$

(4) $$\sigma_{v}=\frac{1}{\rho_{v}}$$

(5) $$\sigma_{m}=\frac{\sigma_{v}}{d_{m}}$$

where:

V = voltage measurement;

I = current;

R = resistance;

T = temperature of the room while recording the voltage measurements;

R_T_ = resistance of the wire at temperature T;

$\rho_{v}$ = resistivity of the wire;

A = cross-sectional area of the wire;

L = length of the wire segment (5 cm);

σ_v_ = electrical conductivity of the wire;

σ_m_ = mass electrical conductivity of the wire.

The mass conductivity of a metal provides the conductivity of the metal after factoring it for the density factor. This is obtained by dividing the electrical conductivity by the density of the metal. At 100% IACS, the mass conductivity of annealed copper at 20 °C was 6524 Sm^2^/kg. The mass conductivity is a useful indicator, since this value compensates for the effective porosity. Metal with a mass conductivity above 100% IACS exhibits higher conductivity, and below this value indicates low conductivity or metal with impurities [16].

## 3. Results

**Figure 1:**
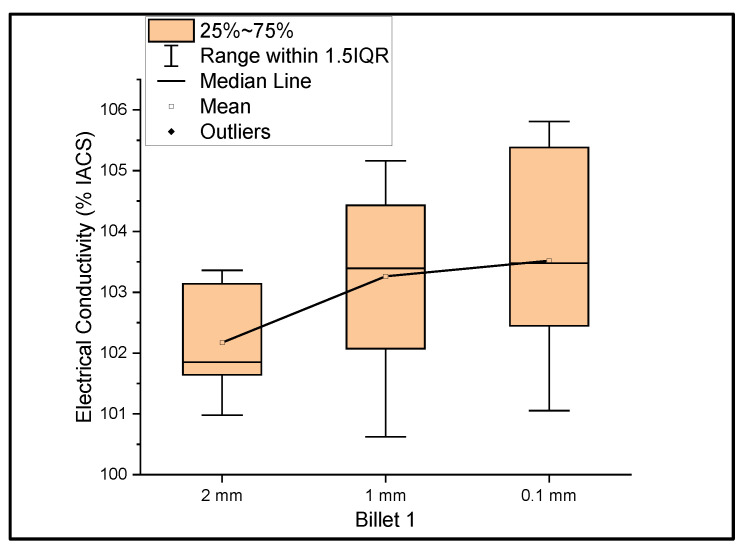
The electrical conductivity of Billet 1 at different cross-sectional sizes.

Figure 1 shows the relationship between the mass electrical conductivity and the size of Billet 1. It was observed that the conductivity of Billet 1 at a 2 mm thickness had a mean of 102.17% IACS and a standard deviation of 0.88. Furthermore, at 1 mm and 0.1 mm thicknesses, the conductivity had a mean of 103.26% and 103.52% IACS with a standard deviation of 1.52 and 1.71, respectively. This graph represents a more accurate representation of the conductivity measurements. The electrical conductivity at 0.1 mm should not be confused with the higher conductivity, but rather the errors due to the thickness of the 0.1 mm ribbon. The thickness of this ribbon is not uniform, and it is a limitation of the rolling process. Moreover, we looked at the big picture to see the feasibility of the rolling operation in producing UCC wire; therefore, not much study has been directed toward producing uniformly thick ribbons.

**Figure 2:**
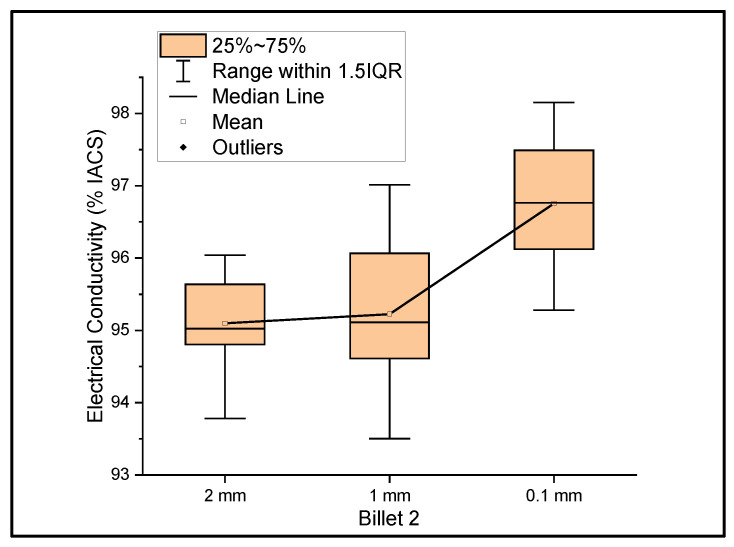
The electrical conductivity of Billet 2 at different cross-sectional sizes.

Figure 2 shows the relationship between the mass electrical conductivity and the size of Billet 2. The measurements were taken over the wires of ribbon thicknesses of 2 mm, 1 mm, and 0.1 mm. The resultant mean conductivity of the wire with decreasing thicknesses was 95.10%, 95.22%, and 96.76% IACS with a standard deviation of 0.69, 1.15, and 1.01, respectively. Although the conductivity values of Billet 2 were lower than those of Billet 1, these values are still within the expected values. Unlike the rest of the billets used as feedstock for rolling operation, Billet 1 was not hot-pressed. The hot-pressed billets consisted of higher amounts of porosity, which was reflected in the conductivity measurements. Billet 2 had 0% CNT precursor material embedded; therefore, the conductivity results from this billet were used as the benchmark for comparison with the other billets with embedded CNT precursor material.

**Figure 3:**
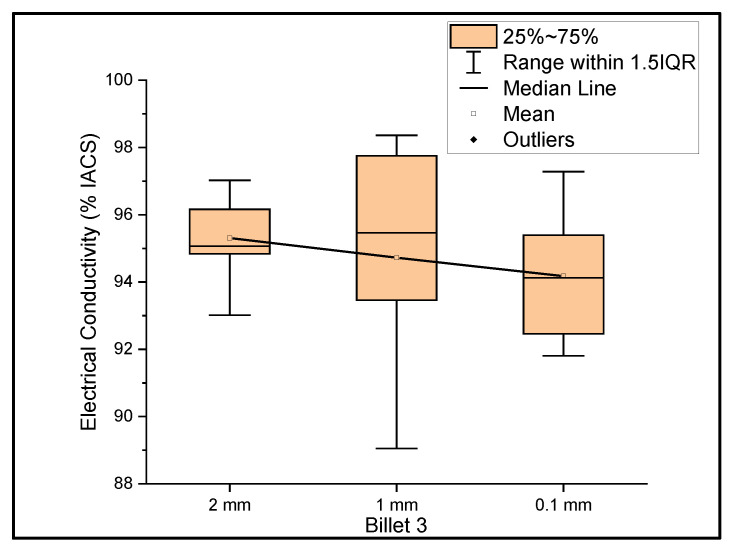
The electrical conductivity of Billet 3 at different cross-sectional sizes.

Figure 3 shows the relationship between the mass electrical conductivity and the size of Billet 3. Billet 3 showed a mean conductivity of 95.31%, 94.72%, and 94.17% IACS with a standard deviation of 1.11, 3.08, and 1.79 for wire of a 2 mm, 1 mm, and 0.1 mm thickness, respectively. Although it looks as if the conductivity decreased with decreasing thickness, it is presumptive to conclude this, since the difference between the mean values was less than 1.5%. Therefore, we considered the conductivity measurements to be uniform at different thicknesses. It was noticed that the results of Billet 3 were close to those of Billet 2. This indicates that the CNT precursor material did not contribute to the overall bulk conductivity and/or there was not a significant amount of the CNT precursor material within the wire to contribute to the electrical conductivity of copper.

**Figure 4:**
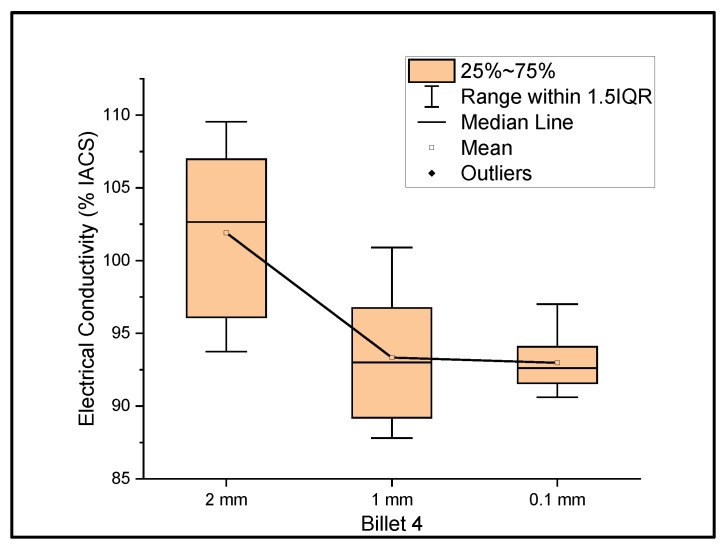
The electrical conductivity of Billet 4 at different cross-sectional sizes.

Figure 4 shows the relationship between the mass electrical conductivity and the size of Billet 4. Billet 4 showed a mean conductivity of 101.91%, 93.33%, and 92.97% IACS with a standard deviation of 5.70, 4.17, and 1.96 for wire of a 2 mm, 1 mm, and 0.1 mm thickness, respectively. The results from Billet 4 show a remarkable increase in the electrical conductivity of the 2 mm wire compared to those of Billet 2 and Billet 3, but upon further rolling, the conductivity dropped to 85% IACS at a 1 mm thickness and 84.68% IACS at a 0.1 mm thickness. This observation shows that the CNT precursor material contributed to the bulk conductivity at a wire thickness of 2 mm, and upon further rolling, the CNTs were either probably destroyed or the ohmic path between the CNTs increased and thereby resulted in lower conductivity, or the CNT acted as an impurity in the copper matrix.

**Figure 5:**
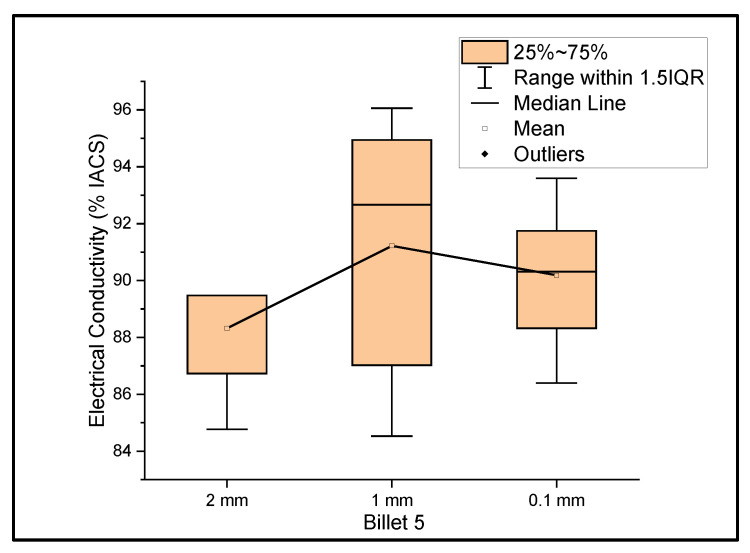
The electrical conductivity of Billet 5 at different cross-sectional sizes.

Figure 5 shows the relationship between the mass electrical conductivity and the size of Billet 5. Billet 5 showed a mean conductivity of 88.32%, 91.22%, and 90.19% IACS with a standard deviation of 1.92, 4.33, and 2.29 for wire of a 2 mm, 1 mm, and 0.1 mm thickness, respectively. The deviation in electrical conductivity from the rest of the billets was clearly observed. The lower conductivity in Billet 5 can be attributed to the improper dispersion of CNT precursor material in the matrix. When rolling the wire from a 2 mm thickness to a 1 mm thickness, the electrical conductivity improved, which can be explained by the breaking of agglomerants and dispersion of CNTs, or looking at the standard deviation, there could be a non-uniform thickness, resulting in an error in determining the thickness.

**Figure 6:**
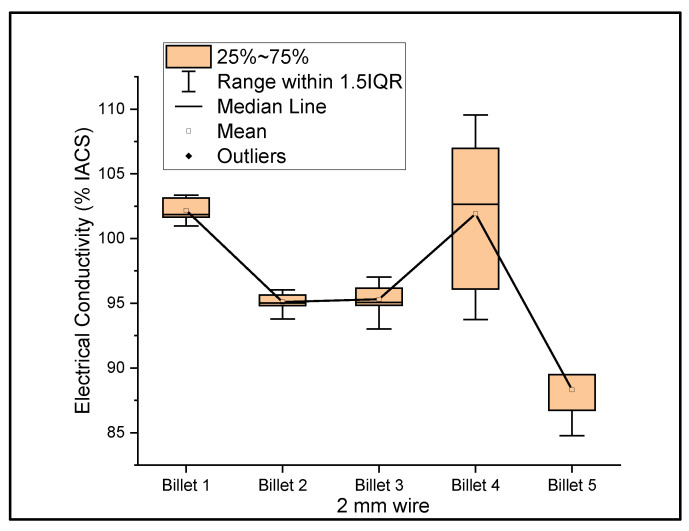
The electrical conductivity of all billets at a 2 mm thickness.

**Figure 7:**
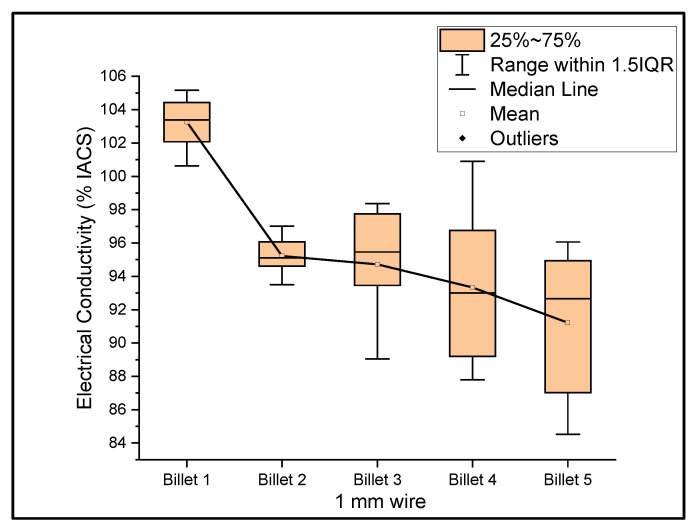
The electrical conductivity of all billets at a 1 mm thickness.

**Figure 8:**
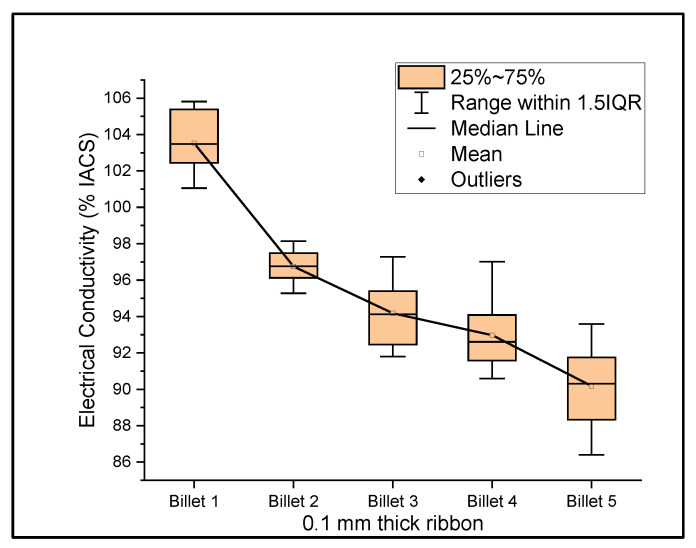
The electrical conductivity of all billets at a 0.1 mm thickness.

Figure 6, Figure 7 and Figure 8 show the graphs that summarize the electrical conductivity results on all billets at different thicknesses. At a 2 mm thickness, the conductivity of Billet 4 is closer to that of Billet 1. This phenomenon is not possible unless the CNTs contribute to the overall bulk conductivity of the wire. In all other cases, the hot-pressed billets failed to exhibit higher conductivities close to Billet 1. It was also noticed that the electrical conductivity decreased as the content of the CNT precursor material increased. Furthermore, Billet 2 with 1/20% (*w*/*w*) of the CNT precursor material showed no significant change in electrical conductivity from Billet 2, indicating that the CNT precursor material is not of a significant quantity to contribute either constructively or destructively to the overall conductivity. The only deviation between Billet 2 and Billet 3 was observed at a 0.1 mm thickness. This could be a result of the errors in determining the thickness of the 0.1 mm-thick ribbon. The probability of this error is high, as we noticed the 0.1 mm thick ribbon’s thickness changes dramatically at different sections of the ribbon. Billet 3 and Billet 4 exhibited decreasing conductivity, which can be attributed to the increasing content of the CNTs. 

## 4. Summary and Conclusions

It is evident from the results that the conductivity of the CNT-embedded copper changed dramatically at different reduction ratios. Although a remarkable improvement in electrical conductivity was not observed, a trend of low- and high-conductive zones in the CNT-embedded copper wire was observed. A consistent conductivity measurement for both oxygen-free copper and hot-pressed billets with no CNT precursor material at various stages of the rolling process was observed. The same effect was not observed on the CNT-embedded copper billets. Although we did not observe high-conductive zones in all of the billets, Billet 4 showed both high- and low-conductive regions. Especially with Billet 4, the conductivity changed dramatically from an average of 101.91% IACS at a 2 mm thickness to 93.33% and 92.97% IACS at 1 mm and 0.1 mm thicknesses, respectively. Even though this is still lower than that of oxygen-free copper, it certainly adds to the excitement for further exploration of UCC wire. Overall, the current method needs improvements at various stages to achieve UCC wire. A more extensive study with collaborators from different fields is required. As of now UCC wire is still in the early stages of scaling up the results.

## 5. Future Works

UCC wire has been gaining attention in recent years, and researchers are investigating feasible methods to achieve UCC wire. There are many areas in our study that need improvement and more thorough research. Some of the areas that need to be focused on based on this study are as follows:

### 5.1. The Effect of Different Grain Sizes of Copper Powder (Raw Material)

This was not investigated in this study, but in future works, it is advisable to compare the results with multiple sizes of copper powder. This study assisted in lowering the porosity of sintered CNT-embedded copper billets.

### 5.2. Using Hot Rolling/Hot Extrusion

This is vital in developing UCC wire. We know that fully cold-worked material tends to exhibit lower electrical conductivity compared to hot-worked and annealed material. In this study, we used cold rolling due to the fact that we had no other options available during the final stage of this overall program. In future work, it is essential to work with either hot rolling/hot extrusion. Extrusion is preferred due to the ability to produce wire/rods by applying immense pressure, which aids in the mechanical bonding of CNTs with copper and greatly assists in maintaining intimate electrical contact between nanotube ends and the matrix material.

### 5.3. Impact of Grain Size on Electrical Conductivity

This study was conducted with an intention to produce commercial-scale UCC wire by investigating favorable manufacturing methods. A rigorous microstructural analysis with respect to how grain size impacts the electrical conductivity needs to be performed.

### 5.4. Determining a Favorable Reduction/Extrusion Ratio

This is possible only if we control the precursor material size. In this study, we are able to observe the effects of the extrusion ratio, as the conductivity of the CNT-embedded copper wire changes drastically with the reduction ratio as the size of the agglomeration’s changes and the ohmic distance between the nanotubes increases.

### 5.5. Amount of CNT Precursor Material Mixed with Copper Powder

Our initial assumption that higher concentrations of CNTs enable additional streaks of ultra-conductive paths was not viable. We faced difficulty in deagglomeration and dispersing CNTs at lower concentrations. Furthermore, the billets with a higher concentration of CNT precursor material fractured in our initial works, which forced us to work with lower concentrations. This is a huge concern, since a lower concentration of CNT might deagglomerate and disperse, but their contribution to bulk conductivity is difficult to measure and their overall contribution can be futile. A rigorous study is required for estimating the optimal concentration of CNT precursor material, the size of CNT, and the electrical conductivity of the wire formed.

## Figures and Tables

**Table 1:** List of Billets and the Concentration of CNTs.

Product	Description
Billet 1	Commercially available O_2_-free Copper
Billet 2	100% copper hot-pressed and rolled
Billet 3	Copper billet hot-pressed with 1/20% MWCNTs
Billet 4	Copper billet hot-pressed with 1/15% MWCNTs
Billet 5	Copper billet hot-pressed with 1/10% MWCNTs

**Table 2:** Theoretical and Measured Densities of the Billets.

Part No.	Theoretical Density (g/cm^3^)	Measured Density (g/cm^3^)
Billet 1	8.96	8.92
Billet 2	8.96	8.53
Billet 3	8.93	8.52
Billet 4	8.92	8.46
Billet 5	8.90	8.37

## Data Availability

The datasets used and/or analyzed during the current study are available from the corresponding author on reasonable request.
